# The Use of 3D Optical Coherence Tomography to Analyze the Architecture of Cyanobacterial Biofilms Formed on a Carbon Nanotube Composite

**DOI:** 10.3390/polym14204410

**Published:** 2022-10-19

**Authors:** Maria J. Romeu, Marta Lima, Luciana C. Gomes, Ed. D. de Jong, João Morais, Vítor Vasconcelos, Manuel F. R. Pereira, Olívia S. G. P. Soares, Jelmer Sjollema, Filipe J. Mergulhão

**Affiliations:** 1 LEPABE—Laboratory for Process Engineering, Environment, Biotechnology and Energy, Faculty of Engineering, University of Porto, Rua Dr. Roberto Frias, 4200-465 Porto, Portugal; 2 ALiCE—Associate Laboratory in Chemical Engineering, Faculty of Engineering, University of Porto, Rua Dr. Roberto Frias, 4200-465 Porto, Portugal; 3 Department of Biomedical Engineering, University of Groningen, University Medical Centre Groningen, Antonius Deusinglaan 1, 9713 AV Groningen, The Netherlands; 4 CIIMAR—Interdisciplinary Centre of Marine and Environmental Research, University of Porto, Terminal de Cruzeiros do Porto de Leixões, Av. General Norton de Matos s/n, 4450-208 Matosinhos, Portugal; 5 Department of Biology, Faculty of Sciences, University of Porto, Rua do Campo Alegre, 4169-007 Porto, Portugal; 6 LSRE–LCM—Laboratory of Separation and Reaction Engineering–Laboratory of Catalysis and Materials, Faculty of Engineering, University of Porto, Rua Dr. Roberto Frias, 4200-465 Porto, Portugal

**Keywords:** marine biofouling, cyanobacterial biofilms, antifouling surfaces, carbon nanotubes, Optical Coherence Tomography

## Abstract

The development of environmentally friendly antifouling strategies for marine applications is of paramount importance, and the fabrication of innovative nanocomposite coatings is a promising approach. Moreover, since Optical Coherence Tomography (OCT) is a powerful imaging technique in biofilm science, the improvement of its analytical power is required to better evaluate the biofilm structure under different scenarios. In this study, the effect of carbon nanotube (CNT)-modified surfaces in cyanobacterial biofilm development was assessed over a long-term assay under controlled hydrodynamic conditions. Their impact on the cyanobacterial biofilm architecture was evaluated by novel parameters obtained from three-dimensional (3D) OCT analysis, such as the contour coefficient, total biofilm volume, biovolume, volume of non-connected pores, and the average size of non-connected pores. The results showed that CNTs incorporated into a commercially used epoxy resin (CNT composite) had a higher antifouling effect at the biofilm maturation stage compared to pristine epoxy resin. Along with a delay in biofilm development, a decrease in biofilm wet weight, thickness, and biovolume was also achieved with the CNT composite compared to epoxy resin and glass (control surfaces). Additionally, biofilms developed on the CNT composite were smoother and presented a lower porosity and a strictly packed structure when compared with those formed on the control surfaces. The novel biofilm parameters obtained from 3D OCT imaging are extremely important when evaluating the biofilm architecture and behavior under different scenarios beyond marine applications.

## 1. Introduction

Marine biofouling causes severe economic and energetic losses, along with critical environmental and ecological consequences. Carbon nanomaterials, including carbon nanotubes (CNTs), graphene, fullerenes, and diamond-like carbon, have been recognized for their antimicrobial and anti-adhesive properties [1]. Due to their remarkable mechanical strength, high thermal conductivity, and structural stability, CNTs are promising nanomaterials for several applications, namely in the industrial, environmental, and medical fields [2,3]. CNTs have already been tested in antifouling formulations to prevent biofouling, mainly to protect ship hulls, as well as in composite materials that come into contact with seawater. Likewise, CNTs have been reported to impact the composition of marine biofilms, as well as the settlement of macrofouling organisms [4,5]. CNTs can be represented as a single rolled-up graphene sheet (single-walled carbon nanotubes, SWCNTs) or a series of concentric graphene sheets (multi-walled carbon nanotubes, MWCNTs) [6]. These carbon nanomaterials exhibit a concentric cylindrical structure with a diameter in the order of nanometers, varying according to the number of walls, and a length of several microns that is extendable by up to a few millimeters (around 4 mm). CNTs are generally incorporated into a polymeric matrix for application in protective coatings, such as polydimethylsiloxane (PDMS) [7], to improve their mechanical strength [8]. The main antibacterial and antifouling mechanisms of CNTs include the disruption of membrane integrity by electrostatic forces between the microbial outer surface and CNTs, leading to membrane oxidation. Moreover, reactive oxygen species generation may directly harm biological molecules of bacteria and/or indirectly prompt DNA destruction [1,9,10]. Although the mechanism behind the antifouling properties of CNTs is still not clear, their length, diameter, surface area, concentration, and treatment time play a significant role in their antifouling and antimicrobial activity [9,10,11]. Regarding CNT concentration, a loss of cell viability was shown to be correlated with increasing SWCNT loading. Remarkably, 5% SWCNTs may reduce biofilm development from *Bacillus anthracis* spores by 81%, while SWCNT concentrations ≥ 20% can inhibit biofilm formation [12]. Likewise, 0.1 wt% SWCNTs can decrease *Escherichia coli* biofilms by 18%, while, with 1 wt% SWCNTs, the biofilm reduction may reach 76% [13]. On the other hand, a study in which different CNT concentrations (0.1, 1, 2, 3, 4, and 5 wt%) were tested to inhibit *E. coli* biofilm development reported an increase in *E. coli* culturability for surfaces with CNT concentrations between 0.1 and 2 wt% compared to bare surfaces, while a decrease was observed for the remaining CNT concentrations [11]. Among these higher CNT concentrations, 3 wt% was the most promising surface for the inhibition of *E. coli* biofilms. Since CNTs’ antimicrobial activity also depends on their dispersion state [14], a lower dispersion may occur at higher loadings (4 and 5 wt%), leading to a lower antimicrobial effect on CNT-based surfaces [11]. Moreover, it has also been reported that introducing small quantities of CNTs into a polymer network can result in a considerable increase in the antibacterial performance of that polymeric matrix [13]. In fact, polyvinyl-N-carbazole (97 wt%) with 3% of SWCNTs demonstrated similar or stronger bactericidal performance than the surfaces consisting of 100% SWCNTs [15].

The attachment by macrofoulers, such as calcareous hard-fouling organisms (barnacles, mussels, and tubeworms) and soft-fouling organisms (non-calcareous algae, sponges, anemones, tunicates, and hydroids), is responsible for the main consequences of marine biofouling. However, the prevention of adhesion and biofilm development by microfoulers such as bacteria, cyanobacteria, and diatoms reduces the progression of biofouling to the next stages. A deeper knowledge of biofilm behavior and how it interplays with the surrounding environment will enable the development of efficient methodologies to control biofouling and mitigate its negative impacts. New imaging technologies, biochemical methods, and molecular biology tools have contributed to the technological development of biofilm science. Optical Coherence Tomography (OCT) is an exciting modality that overcomes the time-consuming and destructive methodologies of biofilm analysis, such as some microscopic techniques. In addition to the tedious sample preparation, most of the relevant microscopic techniques applied to the study of biofilms require the staining of the sample or the use of fluorochromes, which are expensive and can interfere with the local properties of the biofilm [16]. Moreover, some of them provide low-resolution images only covering a small field of view (FOV). OCT presents several advantages over the common microscopic methods since it is a simple and inexpensive technique, does not require sample preparation and/or staining procedures, and allows for the reconstruction of 3D images by *in situ*, non-invasive, and real-time imaging without affecting the biofilm structure [17]. Moreover, OCT can provide images at the mesoscale relatively quickly, and it allows for a great penetration depth, revealing several details of the biofilm structure [18]. Despite all of the advantages of this optical technique, only a limited set of image processing scripts have been specifically developed for processing OCT biofilm images. Furthermore, the analysis of structural parameters obtained from OCT is not advanced when compared, for instance, to microscopy techniques such as Confocal Laser Scanning Microscopy (CLSM). Indeed, there are several software tools and libraries for biofilm image processing from microscopy, including Image Structure Analyzer (ISA) [19], COMSTAT [20], PHobia Laser scanning microscopy Imaging Processor (PHLIP) [21], *bio*Image_L [22], and DAIME [23].

Studies focused on assessing the marine biofouling mitigation effect of CNT coatings can greatly contribute to improving the knowledge regarding the antifouling properties of these promising materials. Moreover, most marine studies focus on unicellular bacteria, and it is pertinent to address additional microfouler organisms such as filamentous cyanobacteria due to their improved stress and predation resistance [24]. Therefore, the main goal of this study was to analyze the potential of CNT-modified surfaces to delay cyanobacterial biofilms, as well as to evaluate their impact on biofilm architecture using an in-depth OCT analysis. CNTs were incorporated in a commercially available polymer, epoxy resin, since it is commonly used to coat the hulls of small recreational vessels [25,26] due to its unique physical, chemical, and mechanical properties, no safety issues, and low cost [27]. Additionally, epoxy composites have demonstrated high durability and resistance to fatigue and UV irradiation [28]. CNT loading (3 wt%) was chosen according to results obtained in previous studies, in which these carbon-based surfaces were tested to inhibit *E. coli* biofilm development in the medical field [11]. Additionally, textural modifications of CNTs were performed by ball milling (BM) treatment over 4 h to enhance its antimicrobial performance [29] by adjusting the CNT length and opening their closed ends to increase the specific surface area [30]. The textural modifications induced by ball-milling treatment proved to be effective in the inhibition of biofilm formation, reducing the amount of biofilm per surface area, biofilm thickness and surface coverage by 31, 47 and 27%, respectively, when compared to surfaces where CNTs were not ball-milled [11].

The specific aim of this study comprises the development of novel analysis parameters obtained from 3D OCT imaging to evaluate the biofilm structure. Since OCT is an *in situ*, non-destructive technique that can be applied to different fields (e.g., marine, medical, and industrial), and the knowledge of biofilm architecture is important to understand all phenomena related to this complex lifestyle, the analysis carried out in this work is extremely relevant. To the best of our knowledge, this is the first study evaluating the impact of CNT-modified surfaces on cyanobacterial biofilm behavior using an in vitro platform that mimics the hydrodynamic conditions prevailing in real marine environments.

## 2. Materials and Methods

### 2.1. Surface Preparation

To assess the surface antifouling performance on cyanobacterial biofilm development, two control surfaces (glass, a commonly submerged artificial surface found on different equipment in aquatic and marine environments, and epoxy resin, a commercially available coating) and a CNT composite were used. The epoxy resin-coated glass was prepared following the protocol described by Faria et al. [31]. Briefly, the epoxy resin was produced by HB Química (Matosinhos, Porto, Portugal) and consisted of a mixture of HB Eposurf 2 resin and HB Eposurf hardener in a ratio of 10:3 (*v*/*v*). To produce the epoxy resin-coated surfaces, 70 μL of epoxy resin was deposited on the top of glass coupons using spin coating (Spin150 PolosTM, Paralab, Porto, Portugal) at 6000 rpm, with increments of 1000 rpm, for 40 s. The surfaces were dried for over 12 h at room temperature and then for 3 h at 60 °C, according to the instructions of the manufacturer.

The methodology for the preparation of the CNT composite surface was adapted from Gomes et al. [11], in which the PDMS matrix was replaced by epoxy resin. Briefly, commercially available pristine MWCNTs (NanocylTM NC3100, Sambreville, Belgium), produced by catalytic chemical vapor deposition with an average length and diameter of 1.5 µm and 9.5 nm, respectively, were used. CNTs were physically modified by BM treatment (Retsch MM200, Haan, Germany) at 15 vibrations s^−1^ for 4 h. After mixing the two epoxy resin portions, 3 wt% of CNTs was incorporated into the epoxy resin mixture since it was shown to be the most effective CNT load to reduce bacterial biofilm formation in previous studies [11]. After that, 70 μL of the composite (3 wt% CNT-BM/epoxy resin) was deposited on the top of the glass coupons using spin coating, as reported above. In previous work [11], our research group showed that bare PDMS surfaces and CNT composites had a similar film thickness (about 30 μm).

### 2.2. Surface Characterization

#### 2.2.1. Water Contact Angle Measurements

The wettability of glass, epoxy resin, and CNT composite surfaces was assessed by measuring the contact angles with water ($\theta_{w}$). This determination was performed using the sessile drop method on an SL200C optical contact angle meter (Solon Information Technology Co., Ltd., Shanghai, China) at room temperature (25 °C), as previously described [32]. At least 25 determinations for each material were made.

#### 2.2.2. Atomic Force Microscopy (AFM)

AFM studies were performed using a Bruker Catalyst microscope in contact mode by a DNP-D cantilever with a spring constant of 0.06 N/m (Bruker, Billerica, MA, USA). The surface roughness was determined from random areas (75 × 75 or 100 × 100 µm^2^) on two coupons of each material at room temperature. Surface roughness calculations and 2D images were made using the NanoScope Analysis software from Bruker. The roughness height parameter determined was the average roughness ($R_{a}$), which gives the average absolute deviation of the roughness irregularities from the mean line over one sampling length [33].

#### 2.2.3. Scanning Electron Microscopy (SEM)

The surface morphology at nanometer resolution was assessed by SEM. All tested surfaces were fixed to SEM stubs using carbon pads (Agar Scientific, UK) and sputter-coated with gold in an SEM coating system (Polaron CVT Ltd., Milton Keynes, UK). The sputter coating conditions were: 5 mA (plasma current), pressure < 0.1 mbar, 800 V, and argon gas for 30 s. The secondary electron detector of a Supra 40VP scanning electron microscope (Carl Zeiss Ltd., Cambridge, UK) was used to obtain the images at an accelerating voltage of 2 kV under different magnifications. Three regions of three different coupons of each material were analyzed.

### 2.3. Organism and Inoculum Preparation

A filamentous cyanobacterial strain, *Nodosilinea* cf. *nodulosa* LEGE 10377, was obtained from the Blue Biotechnology and Ecotoxicology Culture Collection (LEGE-CC) located at the Interdisciplinary Centre of Marine and Environmental Research (CIIMAR), Matosinhos, Portugal [34]. This cyanobacterial strain was previously isolated from a marine sponge at an intertidal zone of Aguda Beach, Arcozelo, Portugal (41.04954 N 8.655339 W). Cyanobacterial cells were grown in 750 mL of Z8 medium [35], supplemented with 25 g/L of synthetic sea salts (Tropic Marin) and vitamin B_12_ (Sigma Aldrich, Merck, Saint Louis, MO, USA). Cultures were grown under 14 h light (10–30 μmol photons m^−2^ s^−1^, λ = 380–700 nm)/10 h dark cycles at 25 °C.

### 2.4. Biofilm Formation

To mimic the hydrodynamic conditions found in marine environments, biofilm formation was evaluated on agitated 12-well microtiter plates (VWR International, Carnaxide, Portugal) under previously optimized conditions for cyanobacterial biofilm development [36]. Firstly, transparent double-sided adhesive tape was placed in the wells to fix the coupons. All coupons and plates were then subjected to UV sterilization, after which the sterile coupons were fixed as previously described [11,36].

Prior to inoculation, the cyanobacterial suspensions were adjusted to a chlorophyll *a* concentration of 0.77 ± 0.03 μg/mL, since chlorophyll *a* quantification is a common method to estimate the biomass in marine environments, and this pigment is unique and predominant in all groups of cyanobacteria [36,37]. Briefly, cells were harvested by centrifugation (3202× *g*, for 5 min at room temperature), the supernatant was discarded and a volume of 2 mL of 99.8% methanol (Methanol ACS Basic, Scharlau Basic, Barcelona, Spain) was added. Then, the cyanobacterial suspension was incubated for 24 h at 4 °C in the dark for maximal chlorophyll *a* extraction, the samples were centrifuged at 3202× *g* for 5 min at room temperature and the supernatant was transferred to a glass cuvette. Absorbance measurements were performed at 750 nm (turbidity), 665 nm (chlorophyll *a*), and 652 nm (chlorophyll *b*) using a V-1200 spectrophotometer (VWR International China Co., Ltd., Shanghai, China). The values obtained were used to calculate the chlorophyll *a* concentration (μg/mL) through Equation (1) [38]:

(1) $$Chl\;a\;\left(\mu g/\mathrm{mL}\right)=16.29\times A^{665}-8.54\times A^{652}$$

These measurements were assessed in triplicate and dilutions were performed using Z8 medium supplemented with 25 g/L of synthetic sea salts and vitamin B_12_. A volume of 3 mL of the respective adjusted cyanobacterial suspension was inoculated in each well. Microtiter plates were then incubated at 25 °C in an orbital shaker with a 25 mm orbital diameter (Agitorb 200ICP, Norconcessus, Ermesinde, Portugal) at 185 rpm, resulting in an average and maximum shear rate of 40 and 120 s^−1^, respectively [36]. Biofilm formation in this system includes the shear rate estimated for a ship in a harbor, 50 s^−1^ [39], and it was shown to predict the biofouling behavior observed upon immersion in the sea for prolonged periods [40]. In order to mimic real light exposure periods, microtiter plates were kept under 14 h light (8–10 μmol photons m^−2^ s^−1^)/10 h dark cycles [36]. Biofilm development was followed for seven weeks (49 days) since a two-month interval for maintenance is the minimum duration for economically viable underwater monitoring systems [36]. During this incubation time, the medium was replaced twice a week.

### 2.5. Biofilm Analysis

Biofilm analysis was performed every seven days, in which two coupons of each surface were analyzed. The culture medium was carefully removed, and the wells were filled with 3 mL of sterile sodium chloride solution (8.5 g/L) [36]. The solution was carefully removed to eliminate loosely attached cyanobacteria. Subsequently, the wells were filled again with 3 mL of sterile sodium chloride solution to evaluate the structure of the cyanobacterial biofilms by OCT. To complement the characterization of cyanobacterial biofilms, the determination of their wet weight was also performed over the seven weeks, and at the end of the experiment, the morphology of cyanobacterial biofilms was evaluated by SEM.

#### 2.5.1. Optical Coherence Tomography (OCT)

**Figure 1:**
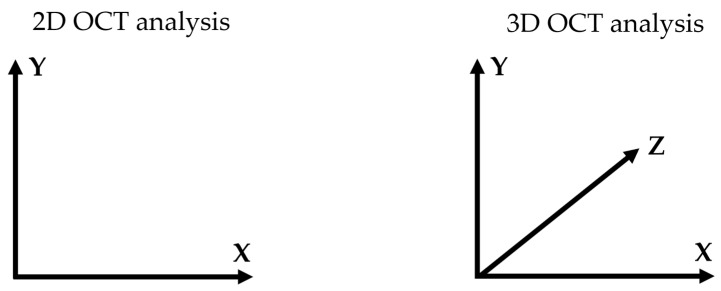
Two-dimensional and 3D coordinate systems for 2D and 3D OCT analyses, respectively. *X* represents the width, *Y* is the height, and *Z* refers to the depth.

Images from cyanobacterial biofilms were captured and analyzed as previously described [36,41]. For each coupon, two-dimensional (2D) and 3D imaging were performed with a minimum of two FOVs to ensure the accuracy and reliability of the obtained results. The 2D and 3D coordinate systems used in this work are shown in Figure 1. For image analysis, the bottom of the biofilm was determined as the best-fitting parabole and hyperboloid, in 2D and 3D images, respectively, that connected the white pixels resulting from light reflection on the substratum surface. A gray-value threshold that separates the biofilm from the background was calculated based on the gray-value histogram of the entire ROI (region of interest) selected [42]. The upper contour line of the biofilm was defined as the pixels with the highest distance to the bottom that had a gray value higher than the gray-value threshold and which were connected to the bottom of the biofilm. Objects not connected to the bottom were rejected from the biofilm structure, and the average biofilm thickness (${\bar{L}}_{F}$) was calculated as a function of the number of pixels (or voxels) between the bottom of the biofilm and the upper contour line for each vertical line in the image $({\bar{L}}_{F}$ is the mean of $L_{F,\;i}$ along the area described as $A_{x,z}$, where $L_{F,\;i}$ is the local biofilm thickness (µm) connected to the bottom at location *i* for each vertical *x,z* column (line of voxels)). In addition to biofilm thickness, novel parameters were analyzed from 3D analysis obtained by OCT, such as the contour coefficient, total biofilm volume, biovolume, volume of non-connected pores and the average size of non-connected pores. For this analysis, the voxel volume of a non-connected pore was defined as about 117 µm^3^, and the standard minimum non-connected pore size in the analysis was 1000 µm^3^. The values of the total biofilm volume, biovolume, and volume of non-connected pores were used as cumulative values along the $L_{F,\;i}$ to the maximum biofilm thickness obtained for each FOV. Consequently, the final values from these parameters were represented as the values achieved at the maximum biofilm thickness. A summary of all variables used in this section is presented in Table 1.

**Contour Coefficient**

The biofilm structure was analyzed by a novel structural parameter—the *contour coefficient*—defined as the number of voxels connected to the background divided by the number of voxels of a horizontal plane, $A_{x,z}$, according to Equation (2):

(2) $$Contour\;coefficient=\frac{1}{N}\overset{N}{\underset{i=1}{\sum }}\frac{C_{F,i}}{A_{x,z}}$$

This parameter enables the analysis of the fraction of the biofilm that is exposed to the surrounding medium. Therefore, values close to 1 reflect a homogeneous and flat biofilm, while in biofilms with heterogeneous structures (e.g., biofilms with streamers), the values are higher than 1.

**Total Biofilm Volume**

The *total biofilm volume* is defined as the number of all connected voxels and non-connected voxels in all images of a horizontal plane multiplied by the voxel size, and it provides an estimate of the total volume of the biofilm (µm^3^) per area of the ROI. The *total biofilm volume* is determined according to Equation (3):

(3) $$Total\;biofilm\;volume\;\left({µm}^{3}/{\mathrm{mm}}^{2}\right)=\frac{\sum_{all\;planes}\left[(Acon_{x,z}+Ancp_{x,z})\times V_{vox}\right]}{A_{ROI}}$$

**Biovolume**

The *biovolume* is defined by the number of all connected voxels in all images of a horizontal plane multiplied by the voxel size, and it provides an estimate of the biomass in the biofilm (µm^3^) per area of the ROI. Biovolume is determined according to Equation (4):

(4) $$Biovolume\;\left({µm}^{3}/{\mathrm{mm}}^{2}\right)=\frac{\sum_{all\;planes}\left[(Acon_{x,z})\times V_{vox}\right]}{A_{ROI}}$$

**Porosity**

The percentage of biofilm *porosity* was quantified from data obtained from the *volume of non-connected pores* and the *total biofilm volume*, according to Equation (5):

(5) $$Porosity\;\left(\%\right)=\frac{Volume\;of\;non-connected\;pores\;}{Total\;biofilm\;volume}\times 100$$

The *volume of non-connected pores* (µm^3^) per area of the ROI is defined as the number of non-connected voxels in all images of a stack multiplied by the voxel size and normalized by area, and it is determined according to Equation (6):

(6) $$Volume\;of\;non-connected\;pores_{x,z}\left({µm}^{3}/{\mathrm{mm}}^{2}\right)=\frac{\sum_{all\;planes\;}\left[Ancp_{x,z}\times V_{vox}\right]}{A_{ROI}}$$

**Average Size of Non-Connected Pores**

The determination of the average size of *non-connected pores* inside the biofilm structure was quantified, assuming a minimum size of *non-connected pores* equal to 1000 µm^3^ and defining a voxel size of 1 as corresponding to 117 µm^3^, as reported above.

#### 2.5.2. Wet Weight Determination

The determination of the biofilm wet weight was performed as previously reported [43]. Briefly, a sterile sodium chloride solution (8.5 g/L) was carefully removed from the wells, coupons were detached, weighed, and the biofilm wet weight was obtained as the difference from the initial coupon weight determined prior to inoculation.

#### 2.5.3. Scanning Electron Microscopy (SEM)

After 49 days of incubation, the morphology of cyanobacterial biofilms formed on the three surface materials was analyzed by SEM. Coupons were removed from the microtiter plates, dehydrated in aqueous solutions of increasing ethanol concentrations (10, 25, 40, 50, 70, 80, 90, and 100% (*v*/*v*)) and left in a desiccator until SEM analysis [44]. The samples were then sputter-coated using the equipment and conditions described in Section 2.2.3 and observed in a Supra 40 VP scanning electron microscope (Carl Zeiss Ltd., Cambridge, UK) at a 2 kV accelerating voltage and a magnification of 1000×.

### 2.6. Statistical Analysis

A total of four replicates (two biological assays with two technical replicates each) were analyzed. Data analysis was performed using the statistical program GraphPad Prism^®^ for Windows, version 6.01 (GraphPad Software, Inc., San Diego, CA, USA). Differences between biofilm wet weight, biofilm thickness, total biofilm volume, biovolume, porosity, and the average size of non-connected pores from data obtained by OCT were evaluated using one-way ANOVA with Tukey’s multiple comparisons test. The error bars shown in the graphs correspond to the standard deviation (SD) of the mean. Statistically significant differences between the different surfaces for the same sampling day were considered for *p*-values < 0.05 (corresponding to a confidence level greater than 95%).

## 3. Results and Discussion

### 3.1. Surface Characterization

It is recognized that surface properties such as topography and physicochemistry affect their antiadhesive and/or antimicrobial behavior [45,46]. Thus, all tested surfaces were first investigated regarding (i) wettability by water contact angle measurements, (ii) topography and roughness by Atomic Force Microscopy (AFM), and (iii) morphology and structure by Scanning Electron Microscopy (SEM). The results obtained from the water contact angle measurements and roughness analysis are shown in Table 2. Given that substrates with a water contact angle ($\theta_{w}$) of <90° are considered to be hydrophilic [47], glass is the most hydrophilic surface ($\theta_{w}$ = 40.9° ± 7.4°), followed by the CNT composite ($\theta_{w}$ = 68.9° ± 4.9°), and the epoxy resin-coated glass ($\theta_{w}$ = 76.3° ± 2.5°), which is significantly more hydrophobic (*p* < 0.05) than the resin composite. Lower hydrophobic properties caused by the incorporation of 3 wt% ball-milled CNTs were also observed in previous work in another polymeric matrix (PDMS) [11]. Regarding the average roughness ($R_{a}$) value determined by AFM (Table 2), glass and epoxy resin appeared to be smoother surfaces ($R_{a}$ of 6.3 and 13 nm, respectively) than the CNT composite, which registered a $R_{a}$ value of about 70 times higher than the remaining surfaces.

**Figure 2:**
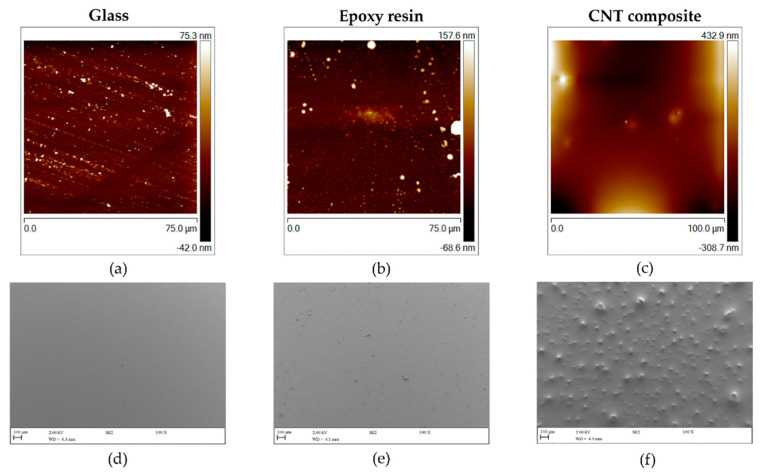
Two-dimensional AFM (**a**–**c**) and SEM (**d**–**f**) images of glass, epoxy resin, and CNT composite. The vertical color bars in the AFM images correspond to the *z*-range (surface height range) of the respective image. The SEM images have a magnification of 100× and the scale bar is equivalent to 100 µm.

Figure 2 reveals the topography and morphology of the tested surfaces obtained from AFM and SEM imaging, respectively. Glass and epoxy resin-coated glass were the most homogeneous and smooth materials (Figure 2a,b,d,e). In opposition, the CNT composite (Figure 2c,f) was the roughest surface, presenting CNT agglomerates that form small elevations on the material (Figure 2f).

### 3.2. Biofilm Formation

**Figure 3:**
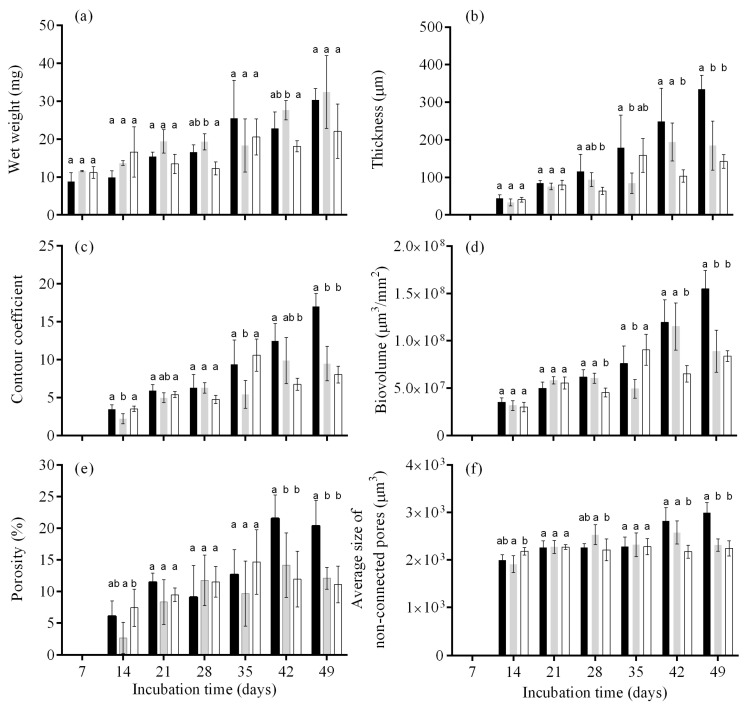
*Nodosilinea* cf. *nodulosa* LEGE 10377 biofilm development on different surfaces (glass—black, epoxy resin—grey, CNT composite—white). The parameters analyzed refer to biofilm wet weight (**a**), thickness (**b**), contour coefficient (**c**), biovolume (**d**), porosity (**e**), and average size of non-connected pores (**f**). Mean values and SD from two biological assays with two technical replicates each are represented. For each sampling day, different lowercase letters indicate significant differences between surfaces (*p* ≤ 0.05; one-way ANOVA with Tukey’s multiple comparisons test).

The structure of biofilms can be numerically quantified with imaging tools to investigate and monitor the effect of different compounds, surfaces and/or environmental factors on biofilm architecture. In the present work, cyanobacterial biofilm development on different surfaces was monitored over 7 weeks and the quantitative results obtained from 3D OCT analysis are shown in Figure 3. The values indicated in Figure 3b–f are only presented from day 14 since, for the first sampling day (day 7), the biofilm thickness was below the OCT range. In general, a gradual temporal increase in biofilm wet weight (Figure 3a), thickness (Figure 3b), and biovolume (Figure 3d) were observed, showing that this filamentous cyanobacterium is a good biofilm former. However, growth was more evident on glass and epoxy resin than on the CNT composite. In fact, for biofilm wet weight (Figure 3a), from day 7 to day 49, increases of 71%, 64%, and 49% were observed for glass, epoxy resin, and the CNT composite, respectively, and for biofilm thickness (Figure 3b), increases of 87%, 82%, and 72% were registered over time for the same surfaces. Regarding biovolume, increases of 77%, 64%, and 64% were observed for glass, epoxy resin and the CNT composite, which indicates that these CNT-modified surfaces can delay biofilm development when compared to pristine epoxy resin and glass. Moreover, on days 42 and 49, a reduction in cyanobacterial biofilm development was observed on the CNT composite surface. Regarding the biofilm thickness, on day 42, the values obtained on glass and epoxy surfaces were 58% and 47% higher than those obtained on the CNT composite surface, respectively, while on day 49, the biofilm thickness was 58% and 23% higher than those attained on the composite (Figure 3b). Additionally, the biovolume obtained on the glass and epoxy surface was 45% and 43% higher when compared to the values obtained on the CNT composite surface, respectively. Moreover, on day 49, these values were 46% and 6% higher as compared to the modified epoxy resin (Figure 3b). Since, in the early stages of biofilm formation (days 7, 14, and 21), all parameters were similar between the surfaces, the results suggest that the CNT composite surface may have a greater antifouling effect on the maturation stage of these cyanobacterial biofilms as compared to the other two types of substrates.

Surface topography plays a considerable role in the way in which marine fouling organisms adhere to surfaces, settle on them, and interact with them [48]. In the nanoregime, the topography can have a robust impact on the wettability of the surface, as well as a direct effect on the contact area available for fouler adhesion. Once more surface contact area is available, more settlement may take place and, consequently, the organisms may be more difficult to remove [49]. Moreover, the surface roughness may also promote an increase in the settlement and adhesion strength of biomolecules, including the proteinaceous adhesives used by many marine organisms [50]. Therefore, rough surfaces present an opportunity for fouling organisms to settle within and between the topographic features, protecting them from hydrodynamic forces [51]. Highly textured surfaces also provide greater surface area for adhesive cements to adhere [52]. Even though topography does not act as a unique mechanism, it is still a key feature that must be considered in the design of new materials. Since, in the early stages of biofilm formation, the values attained were similar between the surfaces (Figure 3), it appears that differences in surface properties, namely in average roughness (Table 2, Figure 2), may have been responsible for the differences registered in the later stages of biofilm development. Therefore, an antimicrobial effect rather than an anti-adhesive effect may explain the impact of CNT-modified surfaces in long-term cyanobacterial biofilm development. CNT composites may lead to unstable and weak biofilm development due to the piercing effect in the cell’s membrane of the first biofilm layers [11]. However, this viability effect may be reflected only in the subsequent biofilm layers, as cell-to-cell adhesion will be hampered if cells in the initial layers are damaged [53]. Moreover, biological processes such as cell reproduction and extracellular polymeric substance (EPS) production by these first-adhered impaired cells may affect biofilm development.

Biofilms formed on the CNT composite were also more homogenous on days 42 and 49 than those developed on glass and epoxy resin (Figure 3c), since the values of the contour coefficients were closer to 1, which reflects a homogeneous and flatter biofilm. Indeed, on day 42, the contour coefficient was around 46% and 32% lower for the CNT composite surface when compared to glass and epoxy resin, respectively. On the other hand, on day 49, these differences reached 53% and 15% when compared to the values obtained on glass and epoxy resin. Since the top of the biofilm grown on the carbon-based surface became flattened, without heterogeneous top structures (such as streamer structures), the superficial area in contact with the surrounding environment decreased, as well as the ability of nutrients and oxygen to penetrate within the biofilm. Likewise, in the maturation stage of biofilm development, a lower percentage of porosity (Figure 3e) and a smaller average size of non-connected pores (Figure 3f) were determined for biofilms formed on the CNT composite surface when compared with control surfaces.

The lower porosity and average size of non-connected pores may contribute to the lower viability of the cells located on the deepest biofilm layers since the internal mass transfer of nutrients to the inner layers of the biofilm may be hindered, contributing to the antimicrobial effect of these surfaces.

**Figure 4:**
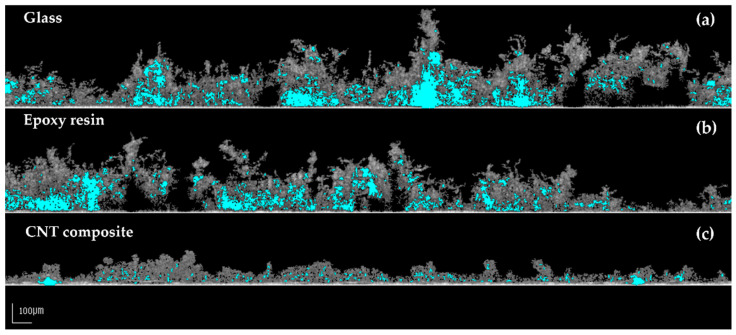
Representative 2D cross-sectional OCT images of *Nodosilinea* cf. *nodulosa* LEGE 10377 biofilms developed on glass (**a**), epoxy resin (**b**), and CNT composite (**c**) after 49 days. The empty spaces in the biofilm structure are filled in blue (scale bar = 100 μm).

**Figure 5:**
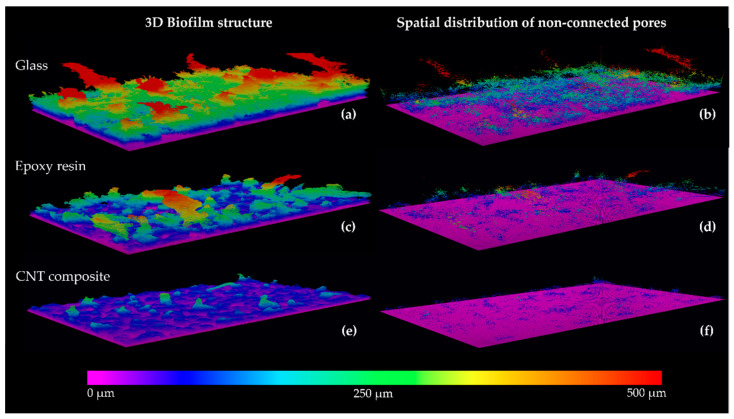
Representative 3D OCT images of *Nodosilinea* cf. *nodulosa* LEGE 10377 biofilms formed on glass (**a**), epoxy resin (**c**), and CNT composite (**e**) after 49 days. The spatial distribution of non-connected pores on the biofilm is also shown (**b**,**d**,**f**). The color scale shows the distance from the substrate surface.

**Figure 6:**
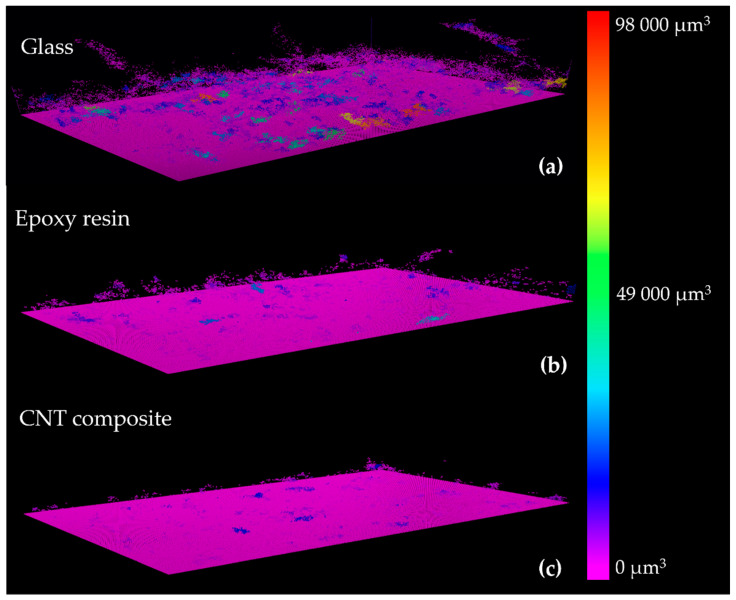
Representative 3D OCT images of the distribution of non-connected pores by size from *Nodosilinea* cf. *nodulosa* LEGE 10377 biofilms formed on glass (**a**), epoxy resin (**b**) and CNT composite (**c**) after 49 days. The color scale shows the range of the size of non-connected pores.

Figure 4, Figure 5 and Figure 6 show representative 2D cross-sectional and 3D OCT images of *Nodosilinea* cf. *nodulosa* LEGE 10377 biofilms on glass, epoxy resin, and CNT composite after 49 days. Both 2D and 3D OCT images illustrate quantitative data on biofilm biomass and porosity (Figure 3). In fact, on the last day of the experiment, a higher percentage of biofilm biomass and porosity was observed on the glass surface when compared with the epoxy resin and the CNT composite surface. The biofilm top structure can also be observed as flatter for biofilms formed on the CNT composite than biofilms developed on glass and epoxy resin (Figure 4 and Figure 5), as it was also indicated by quantitative data (Figure 3c). Moreover, in the biofilm formed on the glass, it was possible to observe long streamers, which can reach around 500 µm (Figure 4). On the other hand, according to representative 3D OCT images, biofilms developed on the CNT composite only present structures that reach around 250 µm. Through representative images of the size of non-connected pores, it was also possible to observe that these values can reach around 98,000 µm^3^ in biofilms developed on glass, while values around 35,000 µm^3^ and 19,000 µm^3^ were achieved on epoxy resin and the CNT composite, respectively.

Mature biofilms consist of multidimensional heterogeneous structures with interstitial pores, which ensure the water and nutrient flow and that influence their resistance to mechanical or chemical challenges [54,55,56,57]. Biofilm biomass, thickness, and structure have a strong effect on the performance of underwater marine devices [58]. Consequently, the knowledge of these biofouling parameters is essential for the design and maintenance of submerged marine equipment. In this work, CNT composite surfaces were tested for the prevention of cyanobacterial biofilms. Biofilms formed on this carbon-modified surface were more homogeneous, flatter, less porous, and had a tightly packed structure compared with the control surfaces, as it can be proved by the quantitative (Figure 3c,e,f) and qualitative (Figure 4, Figure 5 and Figure 6) data. Although biofilm growth is reduced on these surfaces, which may be beneficial for the performance of nautical equipment, it may have an impact on the efficacy of methods to eradicate the biofilms formed on this surface material. Firstly, due to their lower porosity and a smaller average size of non-connected pores (Figure 3e,f), the internal mass transfer may be hampered, and chemical compounds used for biofouling control may not reach the inner layers of the biofilm [59]. Moreover, a homogeneous structure (Figure 3c) can be associated with greater cohesion across the whole biofilm structure. In this scenario, the detachment of biofilm components either by increasing hydrodynamic forces or by applying mechanical cleaning methods would be facilitated if a heterogeneous structure was present (such as the presence of some streamers). As a flatter biofilm was present, the detachment or removal phenomena may be hindered. Although there are several biofilm structural parameters described in the literature [60,61], there has been a tendency to use only a limited number of them [62,63,64]. This evidence makes cross-comparisons difficult and can be justified by the fact that only some of these parameters computed from biofilm images can be intuitively associated with identifiable biological processes. The analysis of biofilms based on biofilm weight, thickness, and biovolume alone, which are typically used for the description of biofilm structure, does not provide complete information on biofilm development. Only the combination of all evaluated structural parameters leads to a complete overview of biofilm behavior on the different surfaces. Moreover, the contour coefficient reported in this work may be a relevant parameter to replace or complement the analysis of biofilm roughness since more reliable structural biofilm information is provided by this novel parameter.

**Figure 7:**
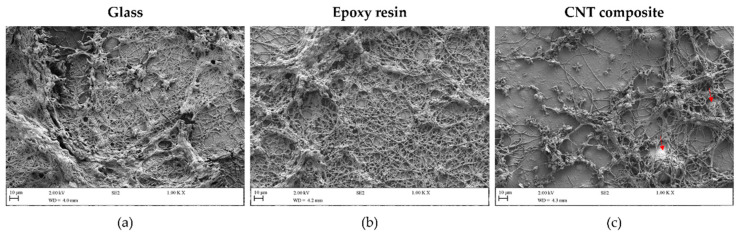
SEM images of *Nodosilinea* cf. *nodulosa* LEGE 10377 biofilms formed on glass (**a**), epoxy resin (**b**), and CNT composite (**c**) after 49 days of incubation. The red arrows in (**c**) indicate clusters of CNTs. Magnification = 1000×; scale bar = 10 µm.

Analysis by SEM was also performed to assess the morphological differences shown by the cyanobacterial biofilms grown on the distinct surfaces (Figure 7). As observed by OCT (Figure 3, Figure 4 and Figure 5), the SEM analysis reveals differences in the cyanobacterial biofilm growth patterns on different surfaces. The highest and lowest cyanobacterial biofilm amounts were observed on glass and the CNT composite surfaces, respectively. Indeed, SEM observations showed that while the biofilm formed on the control surfaces (glass and epoxy resin) looks like a dense filamentous network that covers practically the entire surface area, the biofilm grown on the composite surface presented lower-density cell aggregates.

OCT is a relevant tool for assessing the spatial organization and heterogeneity of biofilm since 2D and 3D datasets contain a representative description of the overall biofilm structure at the mesoscale with µm-resolution [17]. Moreover, by OCT analysis, the laborious nature, as well as the costs entailed with microscopic techniques, can be reduced.

Few studies focus on longer assays for the assessment of CNTs on marine biofilm formation [65]. In fact, some *in vitro* studies have been performed between 6 [66] and 10 days [4], but only a study performed by Xie et al. [67] achieved the 20 days necessary to evaluate the antifouling effect of CNT-based antifouling coatings. Moreover, these studies focus on other marine bacteria, diatoms, algae, and macrofoulers [4,66,67]. For instance, an *in situ* study performed by Sun et al. [68] with pioneer biofilm bacteria over 24 days showed that all CNT/PDMS composites decreased Proteobacteria biofilm formation but increased cyanobacterial biofilm development. Other studies showed a promising reduction in biofilms formed by different microfoulers at different times on distinct CNT-based antifouling coatings [5,45,67,69,70,71,72].

In this study, biofilm was evaluated as a whole structure, including cells, water, and the compounds excreted by the cyanobacterial cells. Considering that a greater number of active cells in a biofilm can lead to greater surface colonization and biofilm development potential, future studies should include complementary techniques to assess the viability and/or the metabolic state of biofilm cyanobacterial cells [73,74]. Since the mechanisms behind the antifouling properties of CNTs are still not clear [2], it is relevant to conduct further assays in marine conditions with CNT-/epoxy resin surfaces, incorporating different CNT concentrations, lengths, diameters, and surface areas, as well as functionalized CNTs, with different polymeric matrices and different fouling organisms [2,9,10,11]. The generation of oxidative stress and mechanical damage through the direct perforation of the microorganisms’ outer membranes and the release of intracellular content are some of the mechanisms that may be involved in CNT action [10]. Indeed, the representative SEM images of the morphology and structure of CNTs in the composite reveal the presence of CNT agglomerates (Figure 2f; Figure S1 in Supplementary Material), which can interact with the membrane of cyanobacterial cells by the known piercing phenomenon and be one of the strategies associated with the antifouling properties of these surfaces. *In situ* assays are also particularly recommended to assess the impact of these surfaces on the adhesion and biofilm formation of multiple microfouling organisms, as well as on the subsequent attachment of macrofoulers. Moreover, additional concerns with these nanostructured surfaces may be related to the fact that they may be damaged in harsh marine environments, leading to a reduction in their antifouling ability and lifespan. Hence, the development of robust and mechanically stabilized surfaces is critical [75].

Different biofilm techniques provide valuable and complementary information about different aspects of the complex biofilm structure. Therefore, a multidisciplinary approach that integrates different methodologies is recommended to obtain a more realistic biofilm representation and to better understand the complex phenomenon of marine biofilm development.

## 4. Conclusions

A set of novel structural parameters obtained from OCT imaging was developed to quantify the marine biofilm structure over time and on three different surface materials, one of them with recognized antifouling activity. CNT-modified surfaces delayed cyanobacterial biofilm development in the maturation stage of the biofilm. Biofilms developed on the composite had reduced wet weight, thickness, and biovolume and were smoother and less porous than those formed on epoxy resin and glass (control surfaces). Analysis of novel parameters obtained by OCT imaging enables a deeper understanding of the biofilm development process in different settings, including the marine environment.

## Figures and Tables

**Table 1:** Variable definition.

Symbol	Description
*x*	Pixel position on the horizontal axis (width)
*y*	Pixel position on the vertical axis (height)
*z*	Pixel position on the perpendicular axis (depth)
*i*	Index of *x,z* position in the horizontal plane
*N*	Number of voxels in the horizontal plane of the region of interest (ROI)
$V_{vox}$	Volume of a voxel (µm^3^)
$L_{F,\;i}$	Biofilm thickness at a given position *i* (µm)
${\bar{L}}_{F}$	Average biofilm thickness (µm)
$A_{ROI}$	Total area of the ROI (mm^2^)
$Acon_{x,z}$	Number of connected voxels identified as belonging to biofilm matrix/bacteria in the biofilm (biovolume) in a horizontal plane at position *y*
$Ancp_{x,z}$	Number of non-biofilm-connected voxels without an open connection to the environment (biofilm holes) in a horizontal plane at position *y*
$C_{F,i}$	Number of voxels identified as belonging to biofilm matrix/bacteria in the biofilm (biovolume) in column *i* (vertical line of voxels at position *i*) and connected to the environment (including corner voxels)

**Table 2:** Water contact angle measurements (*θ_w_*) and roughness (*R_a_*) determined for the tested surfaces.

Surface	Water Contact Angle	Roughness
	$\theta_{w}({}^{\circ})$	$R_{a}(\mathrm{nm})$
Glass	40.9 ± 7.4	6.3
Epoxy resin	76.3 ± 2.5	13.1
CNT composite	68.9 ± 4.9	644

## Data Availability

The data presented in this study are available from the corresponding author upon request.

## Supplementary Materials

The following supporting information can be downloaded at: https://www.mdpi.com/article/10.3390/polym14204410/s1, Figure S1: Representative SEM image of an agglomerate of CNTs on the CNT composite surface. Magnification = 10,000×; scale bar = 1 µm.
