# Supplementary material for: The Use of 3D Optical Coherence Tomography to Analyze the Architecture of Cyanobacterial Biofilms Formed on a Carbon Nanotube Composite
Source: Polymers (Basel). 2022 Oct 19;14(20):4410. doi: 10.3390/polym14204410 (PMC9607013; doi:10.3390/polym14204410)
Supplement: Supplementary file 1 [file polymers-14-04410-s001.zip › polymers-1943403-supplementary.pdf]

Supplementary Material

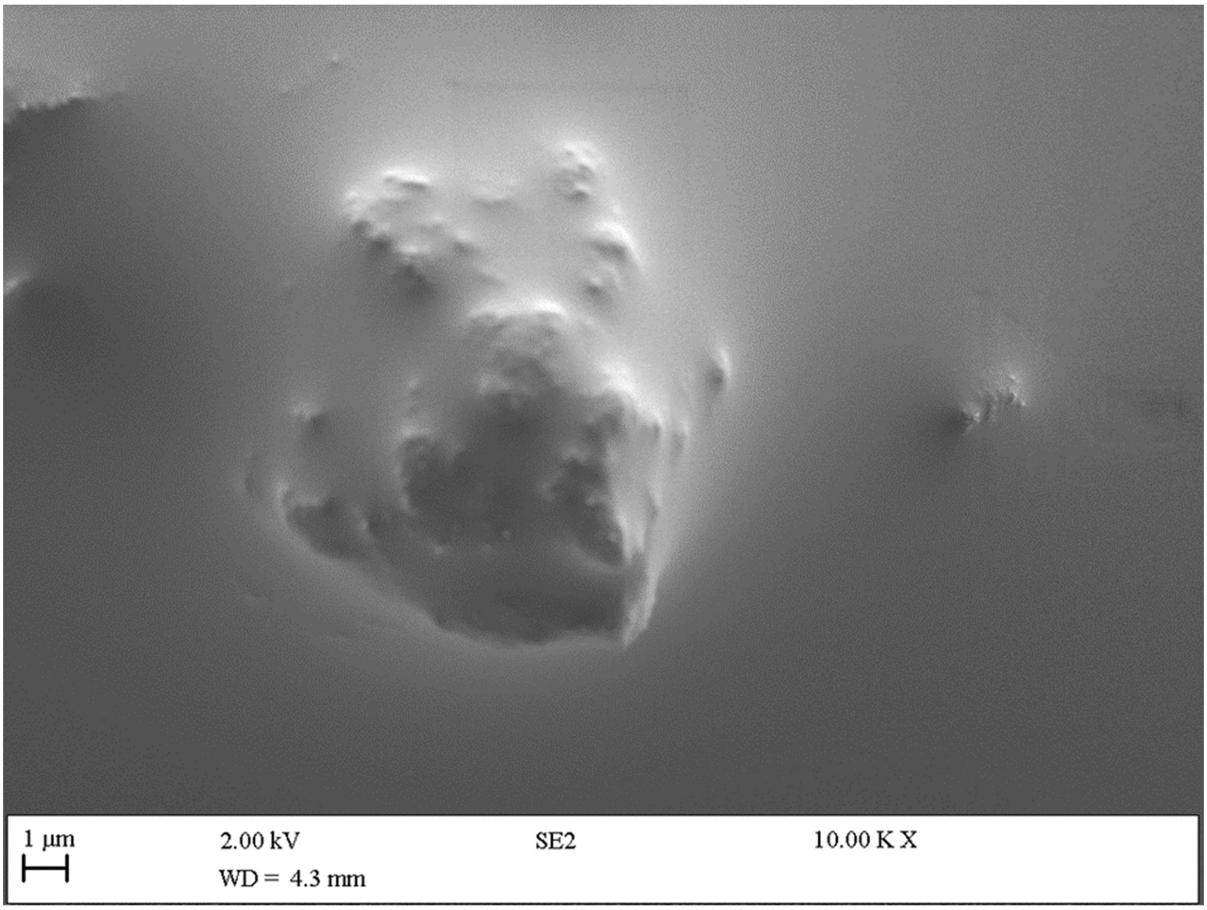

Figure S1. Representative SEM image of an agglomerate of CNTs on the CNT composite surface. Magnification = 10,000×; scale bar = 1 μm.
